# Exploring the Role of *SRC* in Extraocular Muscle Fibrosis of the Graves’ Ophthalmopathy

**DOI:** 10.3389/fbioe.2020.00392

**Published:** 2020-05-08

**Authors:** Mingyu Hao, Jingxue Sun, Yaguang Zhang, Dexin Zhang, Jun Han, Jirong Zhang, Hong Qiao

**Affiliations:** ^1^The Second Affiliated Hospital of Harbin Medical University, Harbin, China; ^2^The Fourth Affiliated Hospital of Harbin Medical University, Harbin, China

**Keywords:** *SRC* gene, extraocular muscle fibrosis, the Graves’ ophthalmopathy, thyroid cancer

## Abstract

The Graves’ disease is an autoimmune disease highly associated with thyroid cancer. The Graves’ ophthalmopathy (GO) is a special Graves’ disease with inflammatory ophthalmopathy being a typical extrathymic complication. GO is caused by the formation of orbital fat and extraocular muscle fibrosis due to the inflammation of orbital connective tissues. Thus, controlling extraocular muscle fibrosis is critical for the prognosis of GO. The objective of this study is to identify and experimentally validate key genes associated with GO and explore their potential function mechanisms especially on extraocular muscle fibrosis. Specifically, we first created a GO mouse model, and performed RNA sequencing on the extraocular muscles of fibrotic GO mice and controls. *SRC* was identified as the most significant unstudied differentially expressed gene between GO mice and controls. Thus, we conducted a few *in vitro* analyses to explore the roles and functions of *SRC* in GO, for which we selected primary cultured orbital fibroblast (OF) as the *in vitro* cell line model. It is known that myofibroblast (MFB), which expresses α-SMA, is an important target cell in the process of fibrosis. Our experiment suggests that TGF-β can induce the transformation from OF to MFB, however, the transformation was inhibited by silencing the *SRC* gene in OF. In addition, we also inhibited TGF-β/Smad, NF-κB, and PI3K/Akt signaling pathways to analyze the interaction between these pathways and *SRC*. In conclusion, the silence of *SRC* in OF can inhibit the transformation from OF to MFB, which might be associated with the interaction between *SRC* and a few pathways such as TGF-β/Smad, NF-κB, and PI3K/Akt.

## Introduction

The Graves’ ophthalmopathy (GO), also called infiltrative exophthalmos, is one type of Graves’ disease with great prevalence (Tsai et al., 2015). About 25–50% of the Graves’ disease patients have varying degrees of GO (Jiskra, 2017). However, the pathogenesis of GO is still unclear. At present, many researches consider it as an autoimmune disease (Burch and Wartofsky, 1993). The symptoms in its early stage mainly include inflammation and edema, while that in the late stage is retrobulbar fibrosis (Heufelder, 1999). Fibrosis of extraocular muscles causes the loss of normal contractile function of muscle tissue, which leads to the limitation of eyeball movement. Patients may suffer from diplopia, strabismus and even compression of optic nerve lead to blindness, which seriously affects their life quality. At present, there is no good treatment for GO and the medication usually cannot prevent the occurrence of advanced extraocular muscle fibrosis. Therefore, it is of great clinical importance to study the pathogenesis of extraocular fibrosis of GO and develop effective prevention and treatment strategies.

Previous studies have suggested that the thyrotropin receptor (TSHR) of orbital fibroblasts (OF), which can regulate thyroid function, plays a pivotal role in GO (Weetman, 2000). In addition to thyroid epithelial cells, TSHR can be detected in extraocular muscle tissue and fat tissue in orbit (Krieger et al., 2016), and the concentration of TSHR in extraocular muscle tissue of GO patients is significantly higher than that of healthy people (Gillespie et al., 2012). Thus, TSHR has been considered as important disease targets in GO (Iyer and Bahn, 2012). The acting mechanism of TSHR is related to various active factors in the process of orbital autoimmune response caused by thyroid orbital autoantigen, which may transform OF to myofibroblast (MFB), a type of cell expressing α-smooth muscle actin (α-SMA; Dik et al., 2016). A few previous studies suggest that the emergence of MFB is the key step in the process of fibrosis (Saika et al., 2016), and the continuous accumulation of MFB or the defect of apoptosis process will lead to the progressive development of fibrosis (Huang and Susztak, 2015).

As another important factor for transforming OF to MFB, transforming growth factor-β (TGF-β) also plays a critical role in the fibrotic diseases of various organs and tissues (Shen et al., 2015). In fact, TGF-β is recognized as the starting hub of the formation and development of fibrosis, which has been widely studied. For example, Steensel et al. (2009) found that the expression level of TGF-β1 mRNA in the orbital tissue of GO patients was twice that of normal people. In addition, TGF-β significantly promotes the proliferation and transformation OF into MFB (Heufelder and Bahn, 1994; Koumas et al., 2003), and regulates the expression of TSHR (Valyasevi, 2001).

At present, researches on GO mechanism are mainly focused on its immunological pathogenesis (Antonelli et al., 2014; Rapoport and McLachlan, 2014; Chen et al., 2015). Recent studies suggest that genes, oxidative stress and other factors may also affect the pathogenesis of GO (Chng et al., 2014; Wang et al., 2015). For example, many genes were abnormally expressed in GO (Chen et al., 2014; Pei et al., 2018), and research shows that gene polymorphism also affects the occurrence and development of GO (Hooshang et al., 2015; Yang et al., 2017). Studies on these aspects can provide a more comprehensive understanding of the pathogenesis of GO. However, a deep exploration on abnormally expressed genes, antioxidant stress, and their acting mechanisms on extraocular fibrosis is more or less ignored, especially at the late stage of the disease. In addition, though it is known that the genetic mechanism of translation and transcription of susceptibility genes in GO patients may cause the self-immune response to TSHR (Brand and Gough, 2010), the mechanism of extraocular fibrosis in the late stage of GO patients has not been clarified. Finally, although it has been found that some molecular mechanisms and signal pathways may be involved in the pathogenesis of GO (Wang, 2014; Tong et al., 2015; Xiao-Ling et al., 2017), the regulation and interaction of these molecular mechanisms need to be further studied, and the role of these molecular mechanisms and signal pathways in GO extraocular muscle fibrosis is unclear.

In this study, we established a GO mouse model by genetic immunization (Sajad et al., 2013), and selected OF as the cell model for *in vitro* study of GO extraocular muscle fibrosis (Lim et al., 2014). Specifically, we first used GO mouse extraocular muscle to screen out key genes for GO extraocular muscle fibrosis, among which *SRC* has not been studied according to literature mining. We then studied the role of *SRC* in GO extraocular muscle fibrosis using the *in vitro* cell model, and its interaction with a few signaling pathways including PI3K/Akt/NF-κB signaling pathway, TGF-β/Smad signaling pathway, and so on. This study provides a new direction for the mechanism of GO development. It also provides a new idea for finding effective intervention targets for the treatment of extraocular muscle fibrosis in GO, which might be of importance clinical significance.

## Materials and Methods

### Mouse Model of Graves’ Orbitopathy

BALB/c female mice (with age 8–10 weeks) were purchased from Animal Experimental Center of Harbin Medical University. Animals were housed under conventional conditions in cages with filter top lids and food made available *ad libitum*. For immunization, BALB/c mice were anesthetized for injection with 50 μL plasmid (1 mg/mL) into each biceps femoris (thigh) muscle. A single injection with the needle entered deep (3–4 mm) into the thigh muscle was performed, with slow release of plasmid into the muscle. Great care was taken to ensure reproducibility of the injection protocol in all immunizations. Injection and *in vivo* electroporation were performed four times at 3-week intervals using an ECM830 square wave electroporator with 7-mm caliper electrodes at 200 V/cm. Application of the current was in ten-20 ms square wave pulses at 1 Hz. All animals were maintained in Specific Pathogen Free and procedures were conducted under Harbin Medical University regulations of accepted standards of humane animal care.

### Cloning and Preparation of Plasmid DNA

The multi-system expression plasmid pTriEx-1.1 Neo was used as a vector and purchased from the BioVector NTCC. Human TSHR A-subunit (amino acid residues 22–289) was amplified for cloning. TSHR A-subunit cDNA region was cloned into *Bam*HI and *Not*I restriction sites in pTriEx-1.1 Neo by amplification from pcDNA3.1-human TSHR plasmid using forward primer 5′-CGCGGATCCATGAGGCGATTTCGGAGG-3′. The cDNA was excised and subcloned into *Bam*HI and *Not*I-digested pTriEx-1.1 NeopTriEx-1.1 Neo vector. The plasmids, termed pTriEx-1.1 Neo-TSHR A-subunit was fully sequenced in strands. All plasmids were grown in *E. coli* XL-1 Blue cells in LB medium in cultures. Purified plasmid concentrations were measured using a spectrophotometer, resuspended at 1 mg/mL in sterile water, and stored at −80°C. Single plasmid preparations were used for the entire set of injections for the group of animals.

### Screening of Differentially Expressed Genes

Three groups of extraocular muscle tissues were entrusted to SeqHealth Tech Co., Ltd., Wuhan, China for RNA Sequencing. The cuff norm was used to quantify the expression levels for each gene normalized by reads per kb of RPKM reads (1). RPKM ≥ 0.5 was defined as a mapped gene. The mapped genes were then used to calculate the difference of RPKM values and the fold changes between GO mice samples and control group samples. A value of *p* < 0.05 and log FC > 1 were classified as a differentially expressed gene (DEG). Map all differentially expressed genes to the various terms of the Gene Ontology database^1^, calculate the number of genes for each term, and then apply a hypergeometric test to find out the difference compared to the entire genome background. Significantly enriched GO entries in the expressed genes. Using Kyoto Encyclopedia of Genes and Genomes (KEGG) Pathway^2^ saliency enrichment analysis, hypergeometric tests were used to find Pathway that was significantly enriched in differentially expressed genes compared to the entire genomic background. After making KO annotations for genes, statistics are based on the KEGG metabolic pathways they participate in. A value of *p* < 0.01 was defined as significant enrichment.

$$\text{RPKM}=\frac{\mathrm{total}\mathrm{exon}\mathrm{reads}}{\mathrm{mapped}\mathrm{reads}\left(\text{millions}\right)\times \mathrm{exon}\mathrm{length}\left(\text{KB}\right)}$$

$$P=1-\sum_{i=0}^{m-1}\frac{\left(\begin{array}{c} m\\ i \end{array}\right)\left(\begin{array}{c} N-M\\ n-i \end{array}\right)}{\left(\begin{array}{c} N\\ N \end{array}\right)}$$

### Cell Culture and Transfections

Primary culture was performed on OFs for BALB/c mice. The cells were cultured in Dulbecco’s Modified Eagle’s Medium (Corning, United States), supplemented with 10% fetal bovine serum (ExCell Bio, Uruguay), and maintained at 37°C in a humidified environment containing 5% CO_2_. For transfection, the cells were plated into 6-well plates with 2.5 × 10^5^cells/well. Once the cells were 70–90% confluent, SRC mimics or NC mimics were transfected into the OFs using Lipofectamine^®^ 3000 reagent (Thermo Fisher Scientific, United States), according to the manufacturer’s protocol. SRC mimics (5′-GCGGCUGCAGAUUGUCAAUTTAUUGACAAUCUGCAGCC GCTT-3′) and its scramble control (5′-ACGUGACACGU UCGGAGAATT-3′) were designed and chemically synthesized by Shanghai GenePharma Co. Ltd. For Inhibitor treatment, 24 h after seeding, the medium was removed and replaced with Oxymatrine (5 mg/mL, cat. no. HY-N0158), JSH-23 (50 μmol/l, cat. no. HY-13982) and PI3K-IN-1 (25 μmol/l, cat. no. HY-12068) or equal amounts of DMSO and incubated for 24h at 37°C. Inhibitors purchased from MedChemExpress, United States. Serum−starved for 24 h, and then treated with human recombinant TGF-β1 (PeproTech, United States). The concentration of TGF-β1 is 10 μg/L and treated for 24 h.

### Immunofluorescent Staining

For Immunofluorescent (IF) staining, the cells grown on the slides were fixed with 4% paraformaldehyde for 30 min at 4°C, then blocked with 5% bovine serum album in for 1 h at room temperature and incubated with primary antibodies overnight at 4°C. The next day, the slides were washed with PBS, and incubated with goat anti-rabbit IgG H&L FICT secondary antibodies (1:1000; cat. no.ab6717; Abcam, United Kingdom) and DAPI (Beyotime Biotechnology, China) for 2 h at room temperature. Fluorescence microscopy images were obtained with a research fluorescence microscope equipped with a digital camera. The following primary antibodies were used: Anti-alpha smooth muscle antibody (1:200; cat. no. ab5694; Abcam, United Kingdom) and Anti-Collagen I antibody (1:500; cat. no. ab34710; Abcam, United Kingdom).

### Real-Time Quantitative PCR

Total RNA was extracted from OFs with TRIzol^®^ reagent (Thermo Fisher Scientific, United States). A total of 1 μg RNA was transcribed into cDNA using transcriptor first Strand cDNA Synthesis Kit (Roche Diagnostics GmbH, Germany) for mRNA according to the manufacturer’s protocol. The expression levels of the genes were detected by qPCR. qPCR was performed using the SYBR Green (Roche Diagnostics GmbH, Germany) dye detection method. The thermocycling conditions were as follows: 95°C for 10 min; followed by 40 cycles of 95°C for 15 s; and 60°C for 60 s. The following primers were used: Acta2 forward, 5′-GACGCTGAAGTATCCGATAGAA-3′ and reverse, 5′-AATACCAGTTGTACGTCCAGAG-3′; Col1a1 forward, 5′-TGA ACGTGGTGTACAAGGTC-3′ and reverse, 5′-CCATCTTTAC CAGGAGAACCAT-3′; Src forward, 5′-CTATGTGGAGCGGA TGAACTAT-3′ and reverse, 5′-ATTCGTTGTCTTCTATGAGC CG-3′; Pik3r1 forward, 5′-AAACAAAGCGGAGAACCTATTG-3′ and reverse, 5′-TAATGACGCAATGCTTGACTTC-3′; Nfkb1 forward, 5′-CAAAGACAAAGAGGAAGTGCAA-3′ and reverse, 5′-GATGGAATGTAATCCCACCGTA-3′; Smad3 forward,5′-AT TCCATTCCCGAGAACACTAA-3′ and reverse, 5′-TAGGTCCA AGTTATTGTGTGCT-3′. Primers were designed and chemically synthesized by Sangon Biotech Co. Ltd., Shanghai, China.

### Western Blotting

Cells were lysed in RIPA buffer supplemented with complete Protease Inhibitor Cocktail tablets (Roche Diagnostics GmbH, Germany) and Phosphatase Inhibitor Cocktail tablets (Roche Diagnostics GmbH, Germany) for 30 min on ice. Protein lysates (30 μg) were subjected to 8% SDS-PAGE (Beyotime Biotechnology, China) and transferred to PVDF membrane. Subsequent to blocking with 5% non-fat milk in 0.05% TBS-Tween-20 (v/v) for 1 h at room temperature, the membranes were incubated with the appropriate primary antibodies overnight at 4°C. The secondary antibodies were horseradish peroxidase (HRP)-conjugated goat anti-mouse IgG (cat. no. ZB-2305; 1:2500; ZSJQ-BIO, China) and HRP-conjugated goat anti-rabbit IgG (cat. no. ZB-2301; 1:2500; ZSJQ-BIO, China). The secondary antibodies were incubated for 1 h at room temperature. Protein detection was performed using an enhanced chemiluminescence substrate (Thermo Fisher Scientific, United States) prior to exposure to film. Primary antibodies used were as follows: Anti-alpha smooth muscle antibody (1:2500; cat. no. ab5694; Abcam, United Kingdom), Anti-Collagen I antibody (1:5000; cat. no. ab34710; Abcam, United Kingdom), TIMP-1 antibody (1:1000; cat. no. NB100-74551; Novus Biologicals, United States), Phospho-NF-κB p65 (ser536)(93H1) Rabbit mAb (1;1000; cat. no.#3033; Cell Signaling Technology, United States), NF-κB p65(D14E12)XP Rabbit mAb (1;1000; cat. no.#8242; Cell Signaling Technology, United States), Phospho-Smad2 (ser465/467)/Smad3(ser423/425)(D27F4) Rabbit mAb (1;1000; cat. no.#8828;Cell Signaling Technology, United States), Smad2/3(D7G7)XP Rabbit mAb (1;1000; cat. no.#8685;Cell Signaling Technology, United States), Anti-PI3 Kinase p85 alpha (phospho Y607) antibody (1:1000; cat. no. ab182651; Abcam, United Kingdom), PI3 Kinase p85 (19H8) Rabbit mAb (1;1000; cat. no.#8242; Cell Signaling Technology, United States).

### Measurement of ROS Level in Cells

The cells (density: 2.5 × 10^5^ cells/well.) were grown in a 6-well plate. After that, DCFH_2__–_ (10 μM) and the culture were combined; they were subjected to incubation for 30 min at 37°C. Warm PBS was used to wash the cells, and generation of reactive oxygen species (ROS) was ascertained from intracellular 2′,7′-dichlorofluorescein (DCF) production that was the result of 2′,7′-dichlorodihydrofluorescein (DCFH_2_) oxidation. A fluorescence enzyme-labeled instrument was employed to determine the level of DCF fluorescence in 488 nm excitation wavelength and 525 nm emission wavelength. ROS kit was purchased from Soleil Technology Co. Ltd.

## Results

### HTSHR A-Subunit Plasmid-Immunized Mice

**FIGURE 1:**
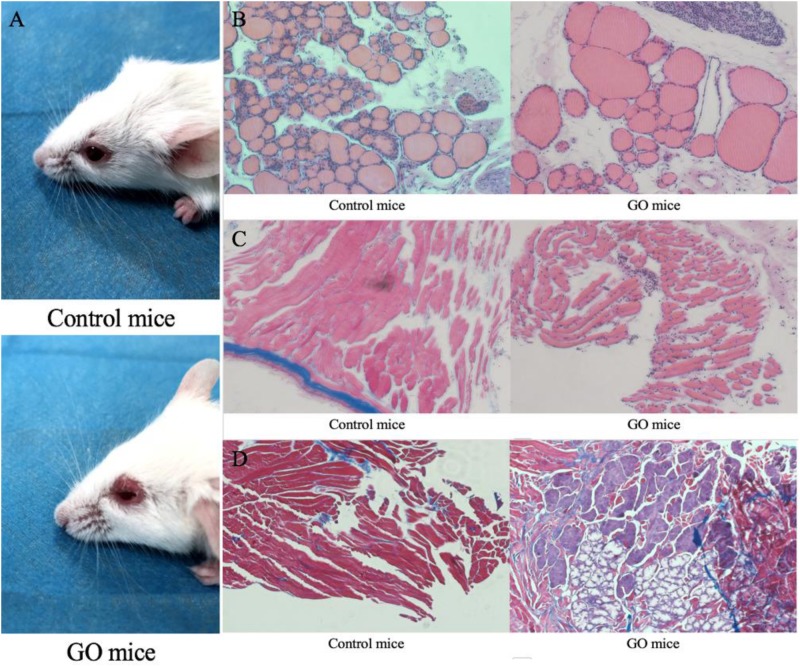
GO mice model and Pathological images. **(A)** control mice and appearance of head region of hTSHR-A subunit plasmid-immunized mouse undergoing chemosis; **(B)** control mice normal thyroid and GO mice hypothyroid gland; **(C)** control mice H&E staining performed normal extraocular muscles and GO mice extraocular muscles showing interstitial inflammatory infiltrate; and **(D)** GO mice Masson’s Trichrome-stained section of orbital muscle to show fibrosis in extraocular muscles and control mice did not show fibrosis.

We initially challenged nine mice with hTSHR A-subunit plasmid by the Moshkelgosha protocol (Sajad et al., 2013). They were weighed weekly before the start of immunization. Animals similarly immunized with pTRiEx1.1 neo (*n* = 9) did not lead to any visible changes in their health or to any histologic manifestations in thyroid and orbital tissue and were used as the control group. As a result, animals in the experiment group showed extraorbital changes with typical signs of acute orbital congestion (chemosis; Figure 1A). The finding of severe sickness prompted us to initially examine thyroid histology by hematoxylin–eosin (H&E) staining, which showed typical pattern of hypothyroidism, with most follicles characterized by thinning of epithelial cells (Figure 1B). In contrast, the H&E examination of thyroid glands of mice in the control group showed normal appearance. We next examined the H&E staining on orbital tissues. While the controlled mice showed normal appearance, the animals immunized with hTSHR A-subunit plasmid showed histologic signs of orbital pathology, and interstitial inflammatory infiltrate into extraocular muscle, which was extended into the muscle tissue and isolating individual fibers (Figure 1C). These symptoms are similar to those described in patients with active GO (Boschi et al., 2005). The hTSHR A-subunit plasmid-*in vivo* electroporation model in female BALB/c mice is recognized for robust antibody responses to TSHR, which persists for months after end of immunization (Zhao et al., 2011). The model therefore gave us the opportunity to evaluate the long-term effect of ongoing anti-TSHR immune response on orbital pathology. Finally, the H&E examination of orbital tissue was characterized predominantly by orbital muscle fibrosis, which by Masson’s Trichrome staining exhibited extensive deposition of glycosaminoglycans with pericellular fibrosis in retrobulbar tissue (Figure 1D). Histologic analysis of the orbital tissue also showed disease heterogeneity in the experiment group with expansion of adipose tissue. None of the animals immunized with control plasmids showed any orbital pathology or disease.

**FIGURE 2:**
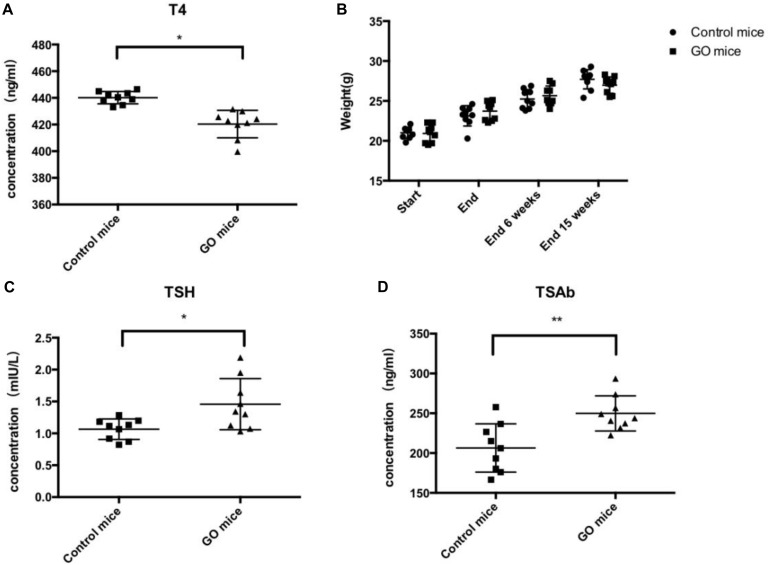
Thyroid function and Antibody. **(A)** control mice and GO mice total serum T4 values; **(B)** control mice and GO mice killed at 6 and 15 weeks after end of immunization; **(C)** control mice and GO mice total serum TSH values; and **(D)** control mice and GO mice total serum TSAb values. *P value < 0.05 compared with control group; **P value < 0.01 compared with control group.

We evaluated thyroid function in the above animals undergoing GO in serum obtained 15 weeks after the end of immunization. Total T4 measurements in mice undergoing experimental thyroid autoimmunity are commonly used for assessment of endocrine status during the course of disease (Gilbert et al., 2006). The animals showed a trend toward lower T4 values, correlating with the findings of hypothyroid glands by histology (Figure 2A). Importantly, the animals in the experiment group showed significant weight gain during the course of immunizations, conferring hypothyroid status (Figure 2B). In addition, the animals showed high levels of TSH (Figure 2C), and the determination of anti-TSHR antibody subtypes showed that the animals are highly positive for TSAbs (Figure 2D).

**FIGURE 3:**
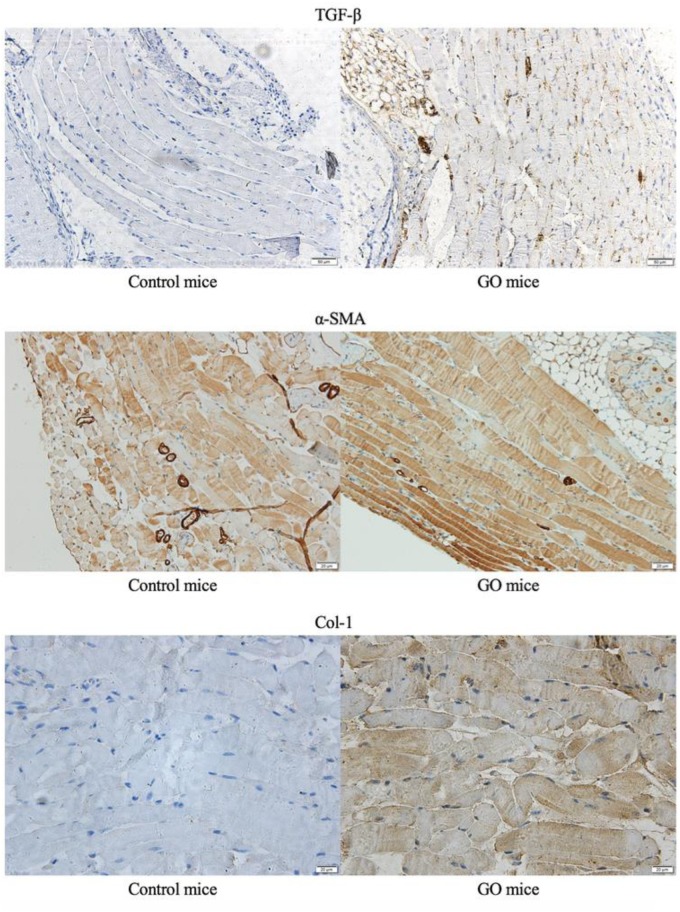
Immunohistochemical staining. The immunohistochemical staining of extraocular muscles in GO mice and control mice.

We performed immunohistochemical staining on the GO mice and control mice. Compared with controlled mice, we detected positive signals on TGF-β, α-SMA and Col-1, and immunohistochemical staining of extraocular muscles for GO mice (Figure 3).

### Screening of DEGs Based on RNA-Seq

**FIGURE 4:**
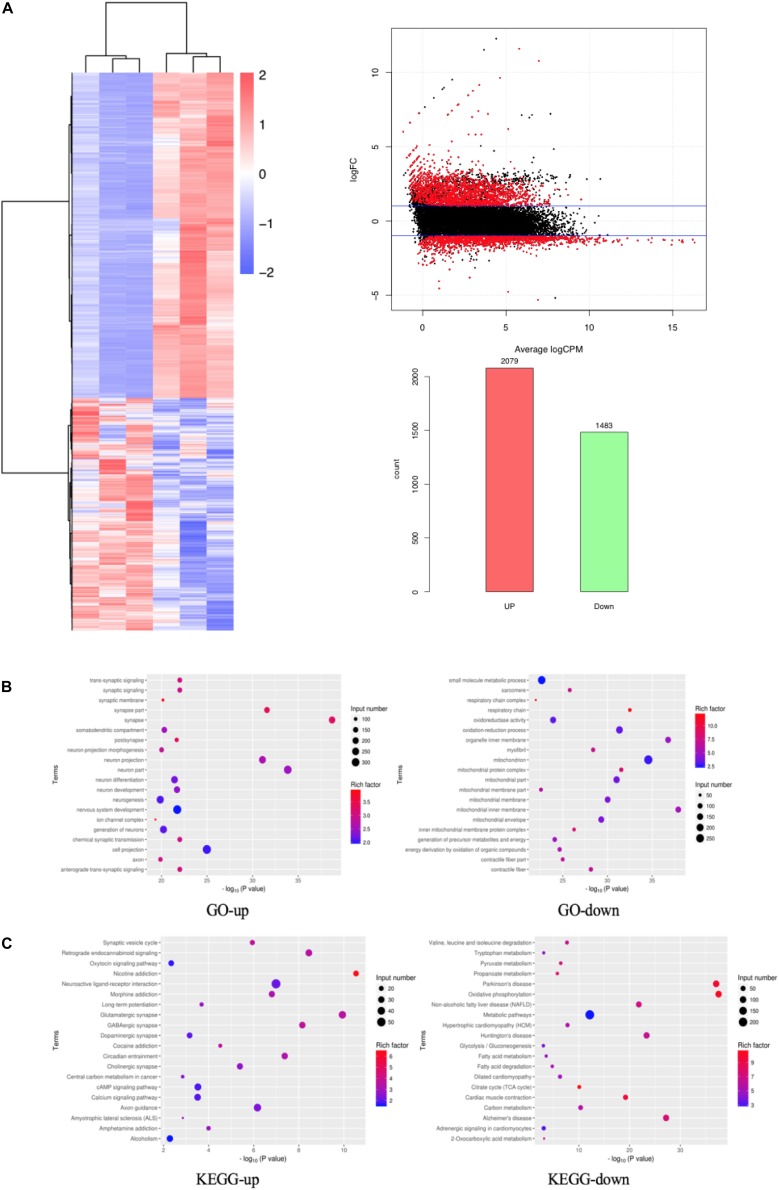
DEGs based on RNA-Seq. **(A)** DEGs shown in a heat map, MA Plot of DEGs and Statistics of DEGs Up-Down; **(B)** GO enrichment map of up- and down-regulated genes; **(C)** KEGG enrichment map of up- and down-regulated genes.

We performed differentially expressed gene (DEG) analysis between GO mice and the controls. Specifically, the extraocular muscle tissues of three GO mice and three control mice were undergone RNA sequencing (RNA-Seq). The genes with transcript per million mapped (RPKM) values <0.5 were removed, and the remaining genes were kept for DEG analysis using DESeq2. A gene with adjusted *p* < 0.05 and | log FC| > 1 was classified as a DEG. A DEG is up-regulated if its logFC > 1 and down-regulated if logFC < −1. Based on this definition, there were 2079 up-regulated and 1483 down-regulated genes. These 3562 DEGs were regarded as candidate genes for further study, and their expression levels were shown in a heat map in Figure 4A. We studied the functions of the DEGs by their enrichment on Gene Ontology terms and KEGG pathways using the hypergeometric test. A term/pathway of *p* < 0.01 was defined as significant enrichment. The significantly enriched Gene Ontology terms and KEGG pathways were shown in Figures 4B,C, respectively. As can be seen from Figure 4B, the top enriched Gene Ontology terms for up-regulated genes include synapse and neuron related function, while the top enriched Gene Ontology terms for down-regulated genes are mainly mitochondria-related functions. Similarly as shown in Figure 4C, the top enriched KEGG pathway for up-regulated genes is nicotine addition, while that for down-regulated genes is oxidative phosphorylation.

### Validation of DEGs by q-PCR

**FIGURE 5:**
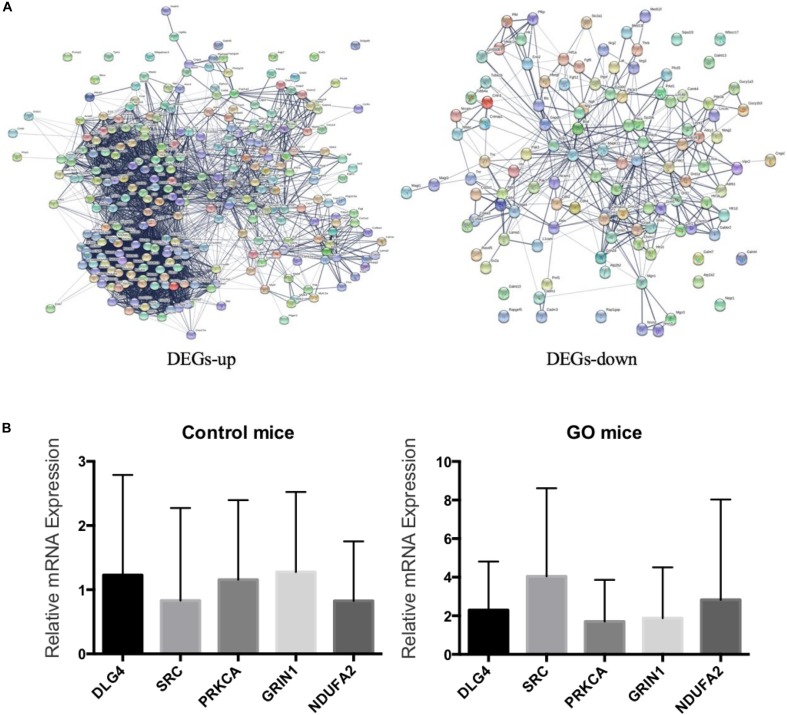
Validation of DEGs. **(A)** Up- and down-regulated genes in PPI; **(B)** Validation of DEGs by q-PCR.

We embedded the 3562 differentially expressed genes into the protein interaction network (STRING), and finally selected five genes including *DLG4*, *SRC*, *PRKCA*, *GRIN1*, and *NDUFA2* with degree greater than 10 (Figure 5A). We then used q-PCR to validate the mRNA levels of five DEGs in extraocular muscle tissues of both GO mice and controls (Figure 5B). As can be seen, the expressions of four genes (*DLG4*, *SRC*, *PRKCA*, and *GRIN1*) were consistent between q-PCR and RNA-Seq, among which we could not find any GO-related research on *SRC* by PubMed search. Thus, we selected *SRC* as the candidate gene for further experiments.

### TGF-β Induces OF and SRC Gene Silencing OF Transformation

**FIGURE 6:**
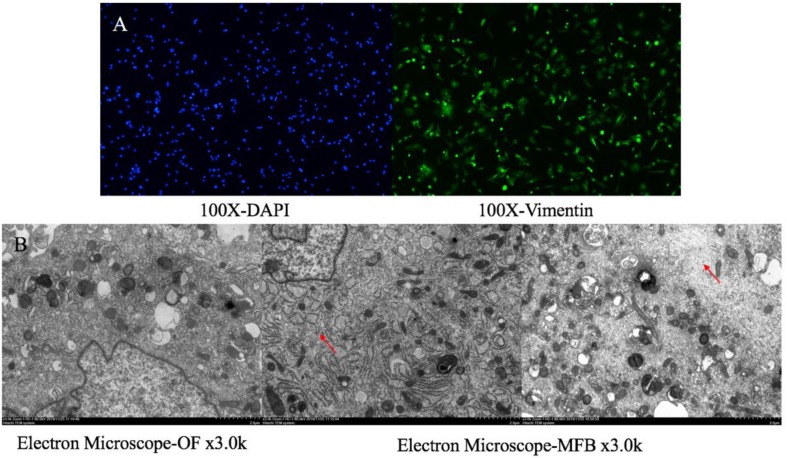
Orbital fibroblasts and myofibroblast. **(A)** Primary culture orbital fibroblasts for BALB/c mice for IF staining; **(B)** OF and TGF-β to induce OF for Electron Microscope.

**FIGURE 7:**
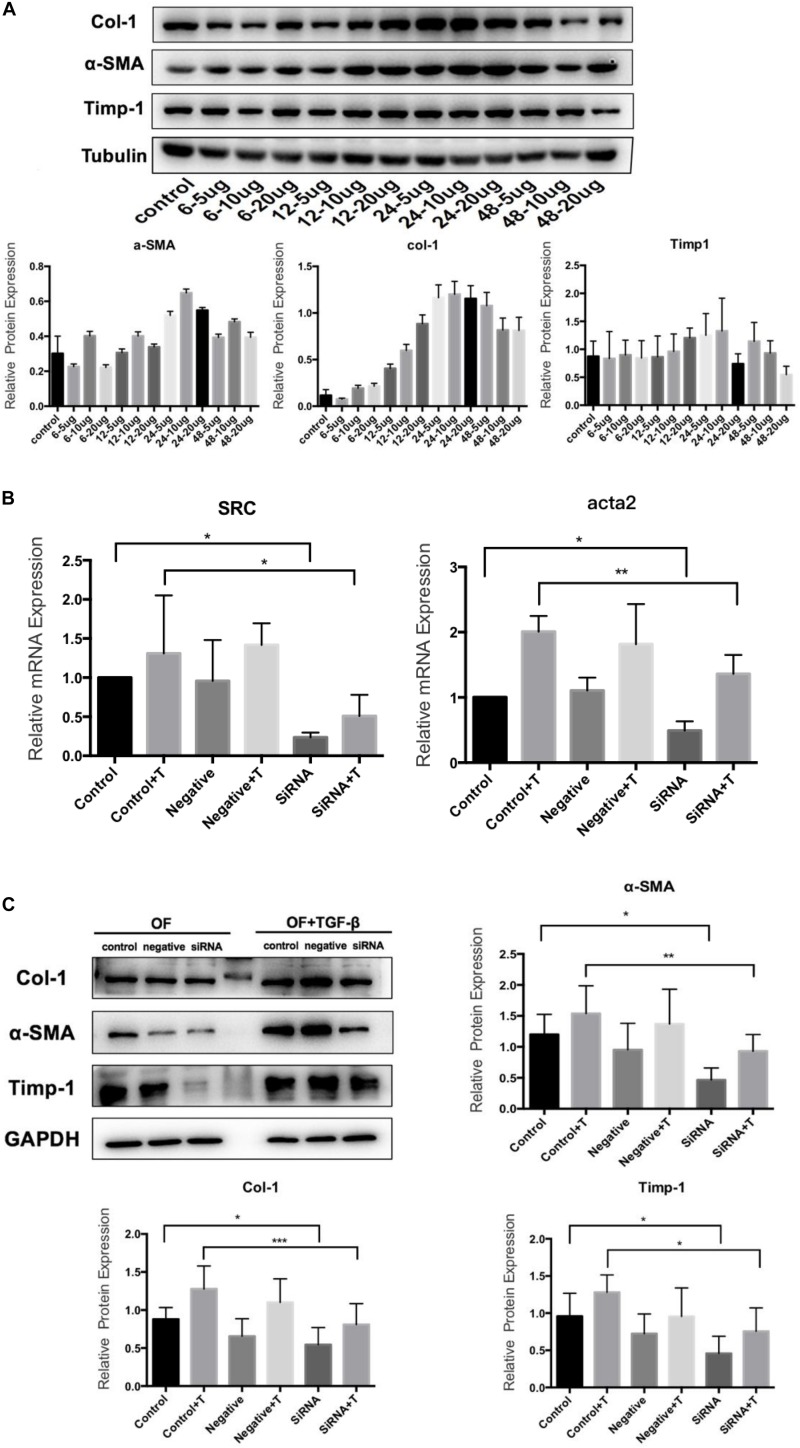
TGF-β1 induces the transformation from OF to MFB. **(A)** Western blot for TGF-β1 concentration and time; **(B)** Src and Acta2 expression in SRC gene knockdown group by q-PCR; and **(C)** the expression levels of α-SMA, Col-1, and Timp-1 in the SRC gene knockdown group by Western blot.

The primary culture of OFs for BALB/c mice was shown in Figure 6. The serum was starved for 24 h, and then treated with human recombinant TGF-β1. We selected 5, 10, and 20 μg/L of TGF-β to induce OF cells for 6, 12, 24, and 48 h respectively, and tested the expression levels of α-SMA, Col-1and Timp-1. The expression was the highest when treated with 10 μg/L for 24 h (Figure 7A), so we chose 10 μg/L TGF-β1 treated for 24 h for further analysis. SRC mimics were transfected into the OFs using Lipofectamine^®^ 3000 reagent, obtaining the OF with the *SRC* gene knockdown. The OF with *SRC* knockdown was further verified by Q-PCR. Furthermore, we can see that *Acta2* expression was also reduced in TGF-b-induced OF with *SRC* gene knockdown (Figure 7B). Western blot results also showed that the expression levels of α-SMA, Col-1, and Timp-1 were reduced in the *SRC* gene knockdown group compared with the control group, and TGF-β-induced OF transformation was inhibited (Figure 7C).

**FIGURE 8:**
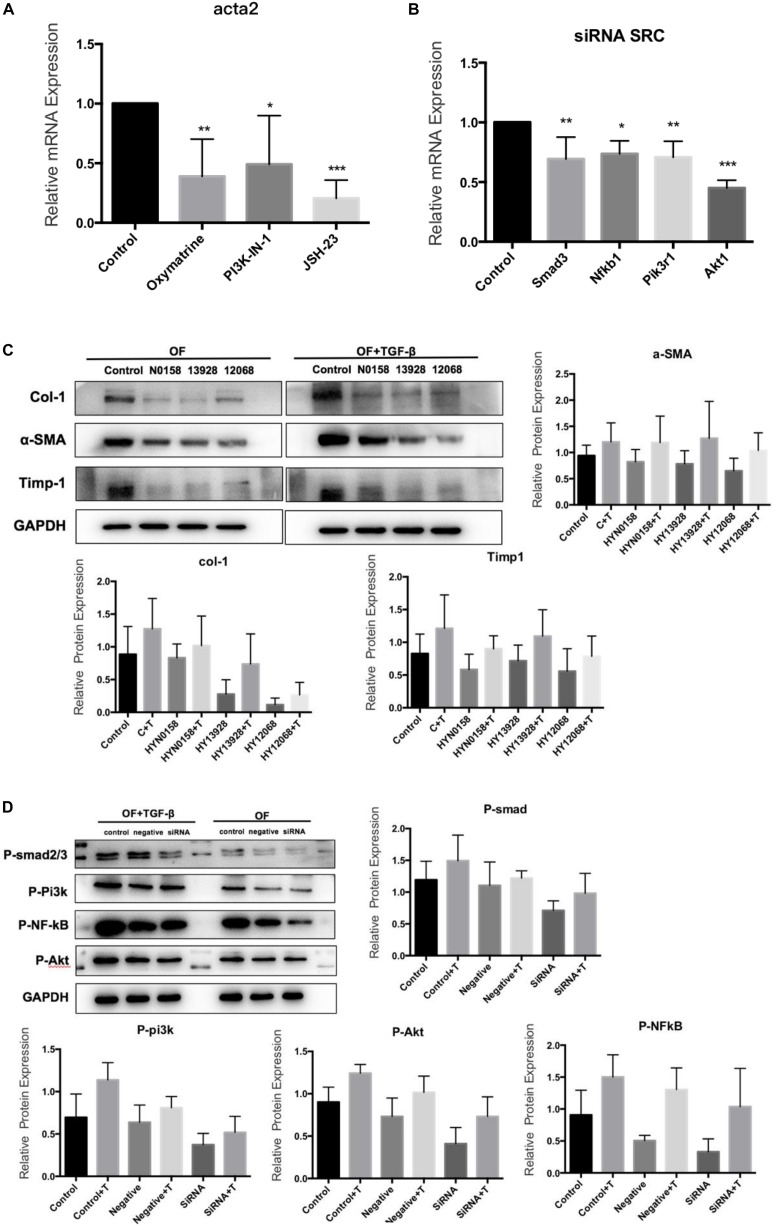
Signaling inhibitors. **(A)** Acta2 expression after Signaling inhibitors by q-PCR; **(B)** the expression of Smad3, Nfkb1, Pik3r1, and Akt1 in the SRC gene knockdown group by q-PCR; **(C)** the expression of α-SMA, Col-1, and Timp-1 after Signaling inhibitors by western blot; **(D)** the phosphorylation of SMAD2/3, NF-κB p65 and PI3K p85 proteins in SRC gene knockdown group by western blot.

For inhibitor treatment, 24 h after seeding, the medium was removed and replaced with the TGF-β/Smad pathway inhibitor Oxymatrine (5 mg/mL), the NF-κB inhibitor JSH-23(50 μmol/L) and the PI3K inhibitor PI3K-IN-1(25μmol/L) to block the TGF-β/Smad, NF-κB, and PI3K/Akt signaling pathways, respectively. And an equal amount of DMSO was used as a control and incubated at 37°C for 24 h. Interestingly, we found that the *Acta2* expression was also reduced after TGF-β induction in OFs after inhibitor treatment (Figure 8A). The Western blot results also showed that compared with the control group, the expression of α-SMA, Col-1, and Timp-1 decreased, and TGF-β induced OF transformation was inhibited (Figure 8C). We further detected the expression of Smad3, Nfkb1, Pik3r1, and Akt1 in the *SRC* knockdown group by q-PCR (Figure 8B). The western blot results also suggested that *SRC* knockdown inhibited the phosphorylation of SMAD2/3, NF-κB p65, and PI3K p85 proteins (Figure 8D).

### ROS Level in Cells

**FIGURE 9:**
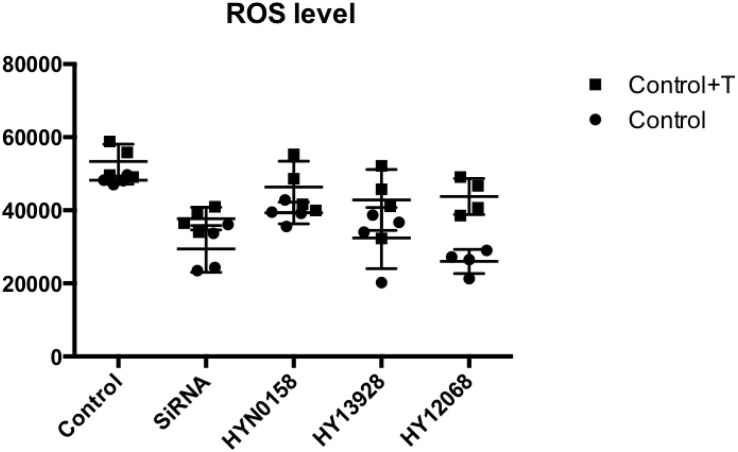
Measurement of ROS level. The ROS generation of each group.

We tested the ROS generation in each group. DCFH_2__–_ (10 μM) and the culture were combined, which were subjected to incubation for 30 min at 37°C. A fluorescence enzyme-labeled instrument was employed to determine the level of DCF fluorescence. As can be seen from Figure 9, the use of inhibitors and *SRC* gene knockdown can significantly inhibit ROS production during TGF-b-induced OF transformation.

## Discussion

We adopted the GO mouse model construction method proposed by Moshkelgosha, which is known to have a high GO mice formation rate of 75% and repeatable (Sajad et al., 2013). Fifteen weeks after the end of immunization, GO mice developed extraocular muscle fibrosis. We then killed the GO mice, obtained the extraocular muscle tissue and thyroid tissue of mice for pathological staining, and detected the serum T4, TSH, TSAb of the mice to evaluate the thyroid function. By comparing with the control group, we determined that the GO mice produced in this experiment had the changes of thyroid function and the fibrosis of extraocular muscles. We identified the differentially expressed genes between the extraocular muscles of GO mice and those of the controls, among which *SRC* was selected as a candidate gene that may cause the extraocular muscle fibrosis. Steensel et al. (2009) showed that the expression of TGF-β1 mRNA in the orbital tissue of GO patients was twice that of normal people. In addition, by detecting the expression level of TGF-β1 in muscle, it was found that the expression level of TGF -β1 in mice with fibrosis was much higher than that in the control group. Thus, it was speculated that TGF-β1 could induce the differentiation of α-SMA protein into MFBs by inducing fibroblasts to express α-SMA, and then induces the occurrence of muscle fibrosis (Liu et al., 2016). Our results confirmed this speculation. Through immunohistochemical analysis, we can see that TGF-β1 expression was higher in extraocular muscle tissue of GO mice in controls. In addition, the expressions of α-SMA and Col-1 were also higher in GO mice than in controls, suggesting that the fibroblast in extraocular muscle of GO mice has begun to change in function and phenotype, and orbital tissue has a large number of extracellular matrix (ECM) accumulation. Moreover, the protrusion of the eyeball can be seen in all GO mice, and the Masson staining of the extraocular muscle can also confirm the fibrosis of the extraocular muscle tissue.

As the target and effector OF autoimmune response, OF can be cultured to serve as an *in vitro* GO model. The transformation from OF to MFB is a key step in the process of fibrosis (Saika et al., 2016), and the expression of α-SMA is a key marker for the transformation (Dik et al., 2016). In this study, we used TGF-β1 to induce the transformation from OF to MFB and to create a cell model of extraocular fibrosis for *in vitro* analyses. The expression of α-SMA was used to evaluate the fibrosis and the expression of COL-1 and TIMP-1 reflected the accumulation of ECM. Our results showed that the OF cell lines without *SRC* knockdown was transformed to MFB under the action of TGF-β1, which expresses a high level of α-SMA, and also produces a high level of ECM. In contrast, the transformation induced by TGF-β1was inhibited with *SRC* knockdown, as indicated by significant low expression of α-SMA, COL-1 and TIMP-1, and less accumulation of ECM. This indicates that SRC gene plays an important role in the fibrosis of go extraocular muscles.

It is worth noticing that we used an inhibitor Oxymatrine to inhibit TGF-β/Smad signaling pathways. In addition to restrain Smad2 and Smad3 phosphorylation, Oxymatrine also restrain the TGF-β1 induced transformation from OF to MFB, as indicated by the low express of α-SMA, Col-1, and TIMP-1. The results confirmed the role of TGF-β/Smad signaling pathway in the process of GO extraocular muscle fibrosis, consistent with the findings of Van Steensel L studies (Steensel et al., 2009). In addition, Smad2 and Smad3 phosphorylation were inhibited in OFs with *SRC* silencing, suggesting that *SRC* might be involved in the functioning of TGF-β/Smad signaling pathway in developing GO.

NF-κB is a kind of nuclear factor, which can promote direct or indirect activation of inflammatory factors, chemokines, inflammatory and TGF-β gene expression (Chen et al., 2012). Thus, it plays an important role in the development of extraocular muscle fibrosis. The results showed that JSH-23, an NF-κB inhibitor, could inhibit the TGF-β1 induced transformation from OF to MFB, which was indicated by relatively reduced expressions of α-SMA, COL-1 and TIMP-1. But the silencing of *SRC* can inhibit the phosphorylation of NF-κB, indicating that *SRC* could play a role in the process of GO extraocular muscle fibrosis by affecting the NF-κB signaling pathway. It is kown that PI3K/Akt signaling pathway plays a key role in TSH induced IL-1ra in GD (Li and Smith, 2014), and it is believed that PI3K/Akt pathway can increase the synthesis of HA by OFs (Xiao-Ling et al., 2017). Therefore, our study tried to determine the relationship between the *SRC* gene and the PI3K/Akt signaling pathway, which is a major upstream component of NF-κB. PI3K/Akt is thought to be involved in the pathogenesis of GO. By using the PI3K inhibitor PI3K-IN-1, we demonstrated that TGF-β1 induced transformation into MFB was also inhibited, with relatively reduced expressions of α-SMA, Col-1 and Timp-1. *SRC* silencing can also restrain the phosphorylation of PI3K and Akt, showing that *SRC* might affect the role of PI3K/Akt signal pathway in the process of external muscle fibrosis.

In addition, previous studies showed that the production of a large number of ROS can activate inflammatory signaling pathways like PI3K/Akt/NF-κB, and promote the expression of type I collagen fibers and TGF-β1, ultimately promoting the occurrence of fibrosis (Cohen-Naftaly and Friedman, 2011; Grochot-Przeczek et al., 2012). Interestingly, *SRC* can positively promote the production of ROS (Nakashima et al., 2017; Walter et al., 2017), and the rise of ROS induced by different stimuli can further promote the activity of *SRC* in cells (Kopetz et al., 2009). On the contrary, antioxidants can inhibit the activation of *SRC* activity by inhibiting ROS production (Fu et al., 2014). In our study, intracellular ROS production was detected, suggesting that the OF with *SRC* silencing produced fewer ROS during TGF-β1 induced transformation than the control group. The results further demonstrated the role of ROS in the process of GO extraocular muscle fibrosis.

Based on the studies, we believe that *SRC* is involved in the ROS mediated oxidative stress process, causing the activation of PI3K/Akt/NF-κB signaling pathway and leading to the occurrence of GO extraocular muscle fibrosis. In addition, *SRC* also plays a role in the development of GO involved in TGF-β/Smad signaling pathway. The results provide a new direction for the study of mechanisms behind GO as well as a potential new intervention target for treating GO patients.

## Data Availability Statement

The data used in this study can be downloaded from https://submit.ncbi.nlm.nih.gov/subs/sra/SUB7185401.

## Ethics Statement

The animal study was reviewed and approved by The Second Affiliated Hospital of Harbin Medical University.

## Author Contributions

HQ conceived the concept of the work. MH, JS, YZ, DZ, JH, and JZ performed the experiments. MH wrote the manuscript.

## Conflict of Interest

The authors declare that the research was conducted in the absence of any commercial or financial relationships that could be construed as a potential conflict of interest.

## Footnotes

1 http://www.geneontology.org/

2 http://www.genome.jp/kegg/
